# The Effectiveness of Antidiabetic Drugs in Treating Dementia: A Peek into Pharmacological and Pharmacokinetic Properties

**DOI:** 10.3390/ijms23126542

**Published:** 2022-06-11

**Authors:** Jiro Ogura, Hiroaki Yamaguchi

**Affiliations:** Department of Pharmacy, Yamagata University Hospital, 2-2-2 Iida-Nishi, Yamagata 990-9585, Japan; hiroaki.yamaguchi@med.id.yamagata-u.ac.jp

**Keywords:** dementia, antidiabetic drugs, pharmacological properties, pharmacokinetic properties

## Abstract

Dementia dramatically affects the activities of daily living and quality of life; thus, many therapeutic approaches for overcoming dementia have been developed. However, an effective treatment regimen is yet to be developed. As diabetes is a well-known risk factor for dementia, drug repositioning and repurposing of antidiabetic drugs are expected to be effective dementia treatments. Several observational studies have been useful for understanding the effectiveness of antidiabetic drugs in treating dementia, but it is difficult to conclusively analyze the association between antidiabetic drug treatment and the risk of developing dementia after correcting for potential confounding factors. Mechanism-based approaches may provide a better understanding of the effectiveness of antidiabetic drugs for treating dementia. Since the peripheral circulation and the central nerve system are separated by the blood–brain barrier, it is important to understand the regulation of the central glucose metabolism. In this review, we discuss the pharmacological and pharmacokinetic properties of antidiabetic drugs in relation to treating dementia.

## 1. Introduction

Dementia is strongly associated with cognitive impairment, behavioral changes, and memory loss; therefore, it dramatically affects the activities of daily living and quality of life [1]. Dementia has multiple social, psychological, physical, and economic impacts on caregivers [1] as well as patients. Therefore, several therapeutic approaches for dementia have been developed; however, an effective treatment regimen is yet to be developed. The development and progression of dementia are associated with several lifestyle habits [2,3], such as smoking [4,5], drinking [6], and metabolic status [3,5,7], including hypertension [8,9] and hyperglycemia [10,11]. Alzheimer’s disease accounts for more than 60% of dementia cases [12], and diabetes is one of the well-known risk factors for dementia [3,13,14,15]. In 1996, a positive association between diabetes and dementia was first demonstrated in the Rotterdam study [15]. In a pooled meta-analysis of over 2.3 million diabetic patients, diabetes was found to be associated with an increased risk for dementia [16]. The risk of dementia has a positive relationship with the severity of diabetes, as indicated by fasting plasma glucose [10] and high HbA1c values [11]. Therefore, drug repositioning and repurposing antidiabetic drugs for dementia are expected to provide affordable and effective treatments for dementia [17].

## 2. Association of Pharmacological and Pharmacokinetic Properties of Antidiabetic Drugs with a Protective Effect on Cognitive Function

The study by Wium-Andersen et al. used one of the largest datasets of diabetic patients in Denmark registered in the National Diabetes Register, spanning from 1 January 1995 to 31 December 2012, and the study was one of the most comprehensive observational studies [18]. This study enrolled 178,403 patients with diabetes and analyzed the association of different types of antidiabetic drugs, as well as their combinations, with the risk of developing dementia. The study demonstrated that patients with diabetes who used metformin, dipeptidyl peptidase-4 (DPP-4) inhibitors, glucagon-like peptide-1 (GLP-1) agonists, and sodium glucose co-transporter 2 (SGLT2) inhibitors had lower odds ratios (ORs) for developing dementia, even after adjustment for confounding factors, including other antidiabetic drug use. A network meta-analysis by Zhou et al. [19] found that patients with diabetes who were treated with DPP-4 inhibitors were associated with a lower dementia risk than those treated with metformin and thiazolidinedione, whereas treatment with insulin was associated with a higher risk. SGLT2 inhibitors were not included in this network meta-analysis.

**Figure 1:**
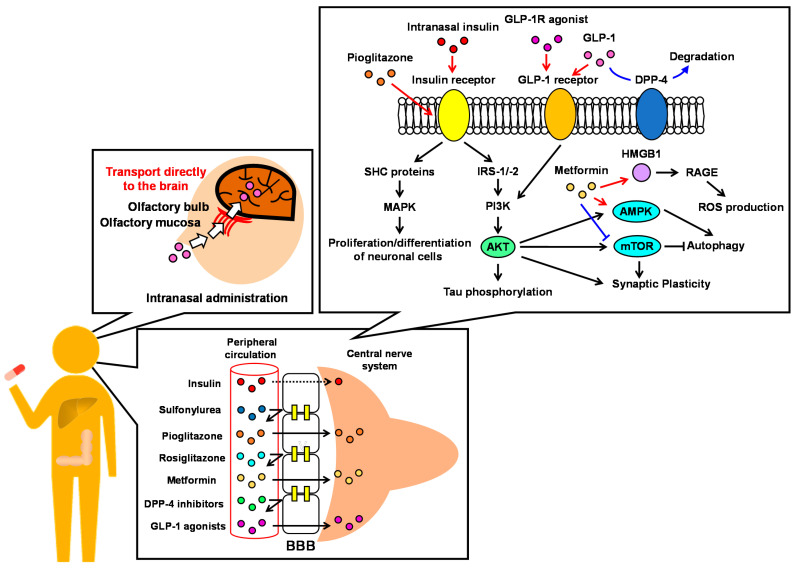
The pharmacological and pharmacokinetic properties of antidiabetic drugs.

These studies are useful for understanding the effective treatment route for dementia using antidiabetic drugs. However, there are several potential confounding factors, such as age, lifestyle, combinations of antidiabetic drugs, treatment period, follow-up period, adherence to antidiabetic medication, and glycemic control status, which make it difficult to correctly analyze the association between antidiabetic drugs, treatment regimens, and the risk of developing dementia through an observational study [20]. Therefore, in this review, we discuss the effectiveness of antidiabetic drugs for dementia, taking into account the mechanisms of effectiveness in terms of pharmacological (Table 1) and pharmacokinetic (Table 2) properties (Figure 1).

### 2.1. The Glucose-Lowering Effect of Antidiabetic Drugs

The peripheral blood circulation and the central nerve system are separated by the blood–brain barrier (BBB). Since glucose transporters are expressed in the BBB [46], glucose can penetrate the BBB. Thus, the glucose-lowering effect of antidiabetic drugs may affect glucose concentration in the central circulation. Acarbose, an α-glucosidase inhibitor, is an antidiabetic drug which is poorly absorbed (only 1–2%) after oral administration [33,47]. Therefore, acarbose does not exert pharmacological effects in the body, and the effect of the drug on cognitive function depends only on its glucose-lowering effect. There are very few studies on the cognitive protective effects of acarbose compared to those of other antidiabetic drugs, but some observational studies have analyzed it. A nested case–control study by Wium-Andersen et al. found that acarbose was not associated with a lower OR for dementia [18]. A population-based cohort study, using Korean National Health Insurance claims data of new-onset type 2 diabetes patients between 2002 and 2013, found that acarbose monotherapy did not reduce the risk of dementia [48]. Moreover, a retrospective cohort study using the longitudinal reimbursement database of Taiwan’s National Health Insurance (NHI) found that α-glucosidase inhibitor use did not reduce the risk of dementia. The cohort included 15,524 matched pairs of ever- and never-users of acarbose from patients with new-onset type 2 diabetes patients between 1999 and 2006 [49]. These findings suggest that the glucose-lowering effect of antidiabetic drugs is insufficient to exert a beneficial effect on dementia.

### 2.2. Increasing Peripheral Insulin Level via Antidiabetic Drugs

Since insulin abnormalities in the central nerve system have been associated with the development of neurodegeneration [50,51], for many years insulin administration was expected to be an effective dementia treatment. Although the saturable transport system of insulin across the BBB was later identified [52], insulin was originally believed to not cross the BBB, and it almost does not penetrate the BBB (AUC_brain:plasma_ = 0.005 after subcutaneous injection) [32]. Therefore, peripherally administered insulin is not a good treatment option. Moreover, some observational studies have found peripheral insulin administration to be ineffective; in some cases, it led to worsening dementia. A prospective open-cohort study using the Swedish Dementia Registry and four Swedish registers/databases found that insulin use was associated with worsening Mini-Mental State Examination (MMSE) scores in both dementia incident and prevalent users [53]. The study was analyzed using the data of 1873 patients including 66 insulin users and 263 non-users. A nested case–control study by Wium-Andersen et al. [18] found that insulin use was ineffective for dementia development after multiple adjustments, although it reduced the risk of dementia in an unadjusted model. Moreover, a Bayesian network meta-analysis included 17 studies containing 1,258,879 individuals, and 3 of the studies with insulin use found that insulin did not reduce the risk of dementia [19]. Sulfonylurea induces insulin secretion by binding to the sulfonylurea receptor in the pancreas. In a Bayesian network meta-analysis, the use of sulfonylurea was associated with a lower risk of dementia than no treatment with antidiabetic drugs [19]; however, the node-splitting analysis found inconsistencies between direct and indirect estimates the risk of dementia in sulfonylurea use. A nested case–control study found that sulfonylurea use was associated with a higher OR for dementia rather than a lower OR [18]. Moreover, a prospective open-cohort study using five Swedish registry databases found that sulfonylurea use was associated with worsening MMSE scores in both dementia incident and prevalent users [53]. These findings suggest that increasing peripheral insulin concentration is not sufficient for causing a beneficial effect on dementia. Both insulin and sulfonylurea did not lower the risk of dementia, instead increasing the risk in some cases. One of the reasons for the ineffectiveness or worsening of the risk for dementia with insulin or sulfonylurea use is the higher risk of hypoglycemia [54,55]. Hypoglycemia is a well-known risk factor for dementia [56,57]. In cohort studies, the dementia risk increased with an increasing number of hypoglycemic episodes, but almost all observational studies analyzing the effect of antidiabetic drugs on dementia risk could not adjust for hypoglycemic episodes as a confounding factor.

Over the last two decades, new roles for sulfonylurea receptors (SURs) in the central nervous system have emerged [58]. SURs itself do not have any functional activity but act as multiple protein complex by binding to subunit proteins such as transient receptor potential melastatin 4 (TRPM4) [58]. The SUR1-TRPM4 channel is upregulated after central nerve system injury and plays a crucial role in the development of cerebral edema [58]. SUR1-TRPM4 is blocked by some sulfonylureas, such as glibenclamide [58]. Moreover, polymorphism of the *ABCC9* gene, which encodes SUR2, is associated with hippocampal sclerosis [59,60]. Unfortunately, to date, no BBB-permeable and clinically used sulfonylureas have been reported. Gliclazide and glibenclamide are widely used antidiabetic sulfonylureas that have poor BBB penetration. The penetration coefficient (*K*_*b*/*p*_) of gliclazide in brain tissue after intra-arterial administration has been reported as 0.12 ± 0.06 [35]. The cerebrospinal fluid-to-plasma and brain-to-plasma concentration ratios of glibenclamide, after intraperitoneal administration in rats, have been reported as 0.0009 and 0.0025, respectively [36]. Glimepiride is another sulfonylurea widely used as an antidiabetic drug; unfortunately, there are no data of its BBB penetration. Therefore, the pharmacological effects of sulfonylurea drugs in the central nerve system cannot be expected.

### 2.3. Intranasal Insulin Administration

As shown above, increasing peripheral insulin concentrations does not exhibit a beneficial effect on dementia; thus, it is important to increase insulin levels in the central nerve system. Intranasal drug administration can avoid interference at the BBB during transfer of the drug to the brain [61]. Moreover, intranasal drug administration reduces the amount of exposure to the drug sustained by the peripheral organs and tissues [61]. This means that the intranasal administration of insulin is able to reduce the risk of hypoglycemia, which explains the ineffectiveness or worsening effect of peripheral insulin administration. Therefore, intranasal insulin administration may have a therapeutic effect in the treatment of dementia. Intranasal insulin administration causes a 2000-fold increase in the AUC_brain:plasma_ ratio relative to subcutaneous injection [32]. In a randomized controlled trial, no cognitive benefits to adults with mild cognitive impairment or Alzheimer’s disease were observed with intranasal insulin treatment compared to placebo over a 12-month period; however, the study execution and the interpretation of results were complicated by issues with the intranasal delivery device [62]. In the prespecified analyses, intranasal insulin treatment using another device was associated with better cognitive function than placebo on the Alzheimer Disease Assessment Scale–Cognitive test (ADAS-Cog12) at 6 months, which strengthened after 18 months of treatment [62]. These findings suggest that intranasal insulin administration may be a potential treatment option for dementia.

### 2.4. Improvement of Insulin Resistance via Antidiabetic Drugs

Insulin receptors are expressed in various regions of the brain [21], and receptor signaling regulates food intake and sympathetic activity [63]. Mechanistically, insulin binds to an insulin receptor and stimulates the phosphorylation of insulin receptor substrates (IRS) [50,51]. The phosphorylated IRS activates the protein kinase B (AKT) pathway, which promotes the translocation of the glucose transporter (GLUT) 4 to the plasma membrane, prevents tau phosphorylation, and induces degradation of misfolded proteins. Moreover, insulin receptor signaling stimulates the phosphorylation of Src homology collagen (SHC)-transforming proteins. The phosphorylated SHC proteins activate the mitogen-activated protein kinases/Rat sarcoma (MAPK/Ras) pathway, resulting in the promotion of the proliferation and differentiation of neuronal cells [50,51].

Thiazolidinediones improve insulin sensitivity by activating peroxisome proliferator-activated gamma-type receptors (PPAR-γs). Since insulin resistance in the brain is strongly associated with the risk of dementia, it is important for neuroprotective thiazolidinediones to penetrate the BBB. Pioglitazone is a widely used thiazolidinedione in clinical practice and exhibits good BBB penetration (712.6 pM ± 231.4 (10.2 mg/kg dose) and 1007 pM ± 273.4 (20.4 mg/kg dose) in the brain after oral administration) [38]. Rosiglitazone is another thiazolidinedione that was used therapeutically before 2007 but is now rarely used because of the associated risk of cardiovascular disease [64]. Rosiglitazone was found to have poor BBB penetration in rodents (only 0.045% inject/g tissue) [39], and limited improvement in learning and memory. In a meta-analysis by Liu et al. [65], pioglitazone was found to be effective in improving cognitive performance using the ADAS-Cog, especially for patients with diabetes, but rosiglitazone was not effective, even for apolipoprotein E (APOE) ε4 non-carriers. The analysis included nine studies with 4327 participants. These findings indicate the importance of BBB penetration of thiazolidinediones for the neuroprotective effects. However, pioglitazone did not delay the onset of mild cognitive impairment in a phase 3 randomized controlled trial (TOMORROW study) [66]. In this study, 3494 patients were enrolled (3061 at high risk for dementia and 433 at low risk) and assigned to pioglitazone (1545 patients; 464 patients discontinued the treatment) and placebo (1516 patients; 640 patients discontinued the treatment) groups. The high-risk participants assigned to the pioglitazone group (2.7%) had the same mild cognitive impairment ratio as the high-risk participants assigned to the placebo group.

### 2.5. GLP-1 Receptor Agonists

GLP-1, an incretin hormone, promotes insulin secretion in a blood glucose level-dependent manner. GLP-1 can penetrate the BBB, although the AUC_brain:plasma_ = 0.052 after intravenous injection is low [67]. GLP-1 is secreted from enteroendocrine L-cells [68]; however, endogenous GLP-1 secreted from the intestine is rapidly degraded by DPP-4. Its half-life is less than 2 min [69]; thus, the beneficial effects of GLP-1 administration are limited in patients with diabetes. However, GLP-1 is also secreted from preproglucagon-expressing neurons in the brain, and GLP-1 receptors are expressed in the brain as well [31]. Although the mechanisms underlying the neuroprotective effects of GLP-1 are not completely clear, the activation of GLP-1 receptor signaling is important for glucose homeostasis in the brain [70]. GLP-1 binds to the GLP-1 receptor in the brain and activates the PI3K/AKT pathway [71,72]. The activation of PI3K/AKT pathways prevents tau phosphorylation, synuclein aggregation, and amyloid β aggregation, which are important pathological changes in neurodegeneration. Simultaneously, GLP-1 receptor signaling increases cellular levels of cAMP and then activates protein kinase A (PKA), resulting in the promotion of proliferation and differentiation of neuronal cells and synaptic plasticity [71,72].

GLP-1 receptor agonists activate GLP-1 receptor signaling, resulting in enhanced insulin secretion. Since GLP-1 receptors are expressed in the brain [31], and receptor signaling regulates glucose homeostasis in the brain, it is therefore important for the agonist to penetrate the BBB to exhibit neuroprotective effects. Although the rates of brain uptake across the BBB for each drug have been found to be different [45], almost all agonists, except for semaglutide and dulaglutide, can cross the BBB. Exendin-4 (also known as exenatide) can cross the BBB in mice after intravenous injection [43], and lixisenatide and liraglutide can cross it after intraperitoneal injection [44], while semaglutide can be detected in the brain after intravenous injection but in negligible amounts [45]. However, there have been no studies on the BBB permeability of dulaglutide. Because of the good BBB permeability of GLP-1 agonists, the beneficial effects of GLP-1 agonists on the risk of dementia have been reported in several observational studies. A study using data from pooled double-blind randomized controlled trials found that GLP-1 receptor agonist users had a lower dementia rate than placebo users [73]. In this analysis, 7907 patients were enrolled in the GLP-1 receptor agonist group and 7913 patients were randomized to the placebo group. A prospective open-cohort study using the Swedish Dementia Registry and four Swedish databases found that GLP-1 receptor agonists were associated with a lower risk of dementia [74]. A nested case–control study by Wium-Andersen et al. [18] found that GLP-1 receptor agonists were associated with a lower OR for dementia. In a small-group randomized controlled trial, liraglutide prevented abnormalities in central glucose metabolism, which are related to cognitive impairment, synaptic dysfunction, and disease evolution [75], although cognitive scores were not significantly different between the liraglutide and placebo groups. In another randomized controlled trial, long-term treatment with dulaglutide reduced cognitive impairment in patients [76]. Considering these findings, GLP-1 receptor agonists may be pharmacologically and pharmacokinetically superior to antidiabetic drugs in maintaining cognitive function. However, further studies are needed to confirm the effect of the GLP-1 agonists because those were introduced relatively recently in clinical settings on the risk of dementia.

### 2.6. DPP-4 Inhibitors

DPP-4 inhibitors prevent the degradation of GLP-1 by DPP-4 and induce insulin secretion. DPP-4 inhibitors, except for teneligliptin and trelagliptin, do not penetrate the BBB, as predicted by ADMET analysis [77]. Teneligliptin and trelagliptin have been experimentally validated to penetrate the BBB [77]. [^14^C]-radiolabeled linagliptin was not detected in the brain after a single intravenous administration in rats [40]. In contrast to in silico analysis, omarigliptin, but not trelagliptin, can penetrate the BBB (brain-to-plasma ratio = 0.23) in rats [41]. Considering these findings, it may be difficult for several DPP-4 inhibitors to permeate the BBB. However, the pharmacological effects of DPP-4 inhibitors could affect cognitive function because GLP-1 can penetrate the BBB [67]. DPP-4 causes a rapid degradation of endogenous GLP-1 secreted from the intestine [69]; thus, if DPP-4 is inhibited by a DPP-4 inhibitor, GLP-1 secreted from the intestine can reach the brain. However, DPP-4 is ubiquitous in the brain [29], and thus, GLP-1 can be rapidly degraded after being translocated into the brain because almost all DPP-4 inhibitors cannot permeate the BBB [40,77]. Some observational studies have shown the beneficial effects of DPP-4 inhibitors on the risk of dementia. An open-cohort study using the Swedish Dementia Registry found that DPP-4 inhibitors were associated with a lower OR for developing dementia and improved MMSE [53]. A nested case–control study by Wium-Andersen et al. [18] found that DPP-4 inhibitors were associated with a lower OR for the development of dementia. Moreover, in a Bayesian network meta-analysis, the use of DPP-4 inhibitors was associated with a lower risk of dementia than no treatment with antidiabetic drugs, and DDP-4 inhibitors were the most effective antidiabetic drugs for dementia [19]. In contrast, two randomized controlled trials of linagliptin [78,79], a non-BBB-penetrating DPP-4 inhibitor, found no association between linagliptin use and maintenance of cognitive function. Unfortunately, there is no randomized controlled trial of BBB-penetrating DPP-4 inhibitors such as omarigliptin. Further pharmacokinetic and clinical studies of individual DPP-4 inhibitors are required to clarify their neuroprotective effects in improving cognitive dysfunction.

### 2.7. Metformin

Metformin exerts multiple pharmacological effects and controls blood glucose levels without increasing insulin secretion. Multiple metformin-targeted proteins include AMP-activated protein kinase (AMPK) [80], mammalian target of rapamycin (mTOR) [81], endosomal Na^+^/H^+^ exchanger (NHE) [82], and high-mobility group box 1 (HMGB1) [83]. AMPK prevents amyloid β aggregation by promoting autophagy [84,85]. In contrast, mTOR inhibits autophagy, resulting in the promotion of amyloid β aggregation [84,85]. Metformin acts as both an AMPK activator and an mTOR inhibitor, thus preventing the development of neurodegeneration. HMGB1 acts as an inflammatory mediator by binding to the receptor for advanced glycation endproduct (RAGE) and toll-like receptor (TLR) 4 and subsequently inducing neurodegeneration [86]. Metformin can directly bind to HMGB1 and suppress HMGB1-mediated inflammation [87]. Therefore, the pharmacological effects of metformin on the brain are important for maintaining cognitive function. Metformin can rapidly cross the BBB (brain to plasma ratio = 0.99, 6 h after a single oral administration) [37], suggesting that metformin is expected to have a beneficial effect on the risk of dementia. However, the effects of metformin on cognitive function remain unclear. In a meta-analysis by Campbell et al., metformin users had a lower risk of dementia and Alzheimer’s disease [88]. The analysis included seven cohort studies, four cross-sectional studies, two randomized controlled trials, and one case–control study. In contrast, in another meta-analysis by Tabatabaei Malazy et al., metformin did not significantly improve cognitive function or protect against dementia [89]. The study included 3 controlled trials and 16 observational studies (cross-sectional and cohort studies). Moreover, while a few randomized controlled trials have been conducted [90,91], the effect of metformin on cognitive function remains controversial.

### 2.8. Sodium Glucose Co-Transporter 2 (SGLT2) Inhibitors

As the name implies, SGLT2 inhibitors suppress the reabsorption of glucose from renal tubules by inhibiting SGLT2, resulting in a decrease in blood glucose level. As SGLT2 is not expressed in the brain [30], the therapeutic benefit of SGLT2 inhibitors on cognitive function may be considered only as a peripheral pharmacological effect. In contrast, some SGLT2 inhibitors have been predicted to inhibit acetylcholinesterase [92,93], which is a therapeutic target for Alzheimer’s disease. Hence, it may be important that the neuroprotective effects of SGLT2 inhibitors penetrate the BBB. Since SGLT2 inhibitors are lipid-soluble and cross the BBB [42], they may show therapeutic benefits for cognitive function. A nested case–control study by Wium-Andersen et al. [18] found that SGLT2 inhibitors were associated with a lower OR of dementia. Moreover, Mui et al. [94] reported that SGLT2 inhibitor users had a lower incidence of dementia than DPP-4 inhibitor users in a retrospective cohort study of patients with diabetes using SGLT2 or DPP-4 inhibitors from 1 January 2015 to 31 December 2019. The study cohort enrolled 13,276 SGLT2 inhibitor users and 36,544 DPP-4 inhibitor users, with a mean follow-up period of 472 days. In a small randomized controlled trial, SGLT2 inhibitors were found to have no difference in cognitive decline compared with liraglutide or DPP-4 inhibitors, suggesting that they may have equivalent benefits [95]. This randomized controlled trial enrolled 39 elderly participants (23 men and 16 women) with diabetes. Based on these findings, SGLT2 inhibitors may be pharmacologically and pharmacokinetically superior to antidiabetic drugs in maintaining cognitive function. However, the target proteins of SGLT2 inhibitors in the brain have not been completely clarified; thus, further studies on the effects of SGLT2 inhibitors on the risk of dementia are needed.

## 3. Summary and Future Prospective

Overcoming dementia has long been a global clinical challenge; however, no effective treatment has yet been established. Drug repositioning and repurposing antidiabetic drugs for dementia are two of the most interesting topics regarding effective treatment of dementia. Indeed, some antidiabetic drugs have shown potential for effective treatment of dementia. Antidiabetic drugs were originally developed to act as regulators of peripheral glucose metabolism; however, for dementia, it is important to understand the central regulation of glucose metabolism because the peripheral circulations and central nerve system are tightly separated by the BBB. Evidence of the pharmacological effects on the brain and BBB permeability of anti-diabetes drugs were gradually being incresing, but not all of these effects have been revealed.. If the pharmacological and pharmacokinetic properties of antidiabetic drugs are clarified, and clinical trials based on these properties are conducted in the future, a society that can overcome dementia may be realized.

## Figures and Tables

**Table 1:** Antidiabetic drugs that target proteins and their brain expressions.

	Pharmacological Effect	Target Protein	Brain Expression	Ref.
Insulin	Activates insulin receptor signaling	Insulin receptor	Yes	[21]
α-Glucosidase inhibitors	Suppress glucose absorption	α-Glucosidase	Yes	[22]
Sulfonylureas	Promote insulin secretion	Sulfonylurea receptor	Yes	[23]
Metformin	Improves insulin sensitivity, etc. *	AMPK, etc. *	Yes	[24,25,26,27]
Thiazolidinediones	Improve insulin sensitivity	PPARγ	Yes	[28]
DPP-4 inhibitors	Prevent GLP-1 breakdown	DPP-4	Yes	[29]
SGLT2 inhibitors	Promote glucose excretion	SGLT2	No	[30]
GLP-1 receptor agonists	Activate GLP-1 receptor signaling	GLP-1 receptor	Yes	[31]

*: The pharmacological effect and target proteins of metformin have not yet been completely identified.

**Table 2:** The BBB permeability of antidiabetic drugs.

	Drug	Species	Dose	Administration Route	Plasma Level	Brain Level	Plasma/Brain	Ref.
Insulin	Subcutaneous insulin	Mouse	2.4 IU	Subcutaneous injections	AUC_0-t_ = 520,351 h·μIU/mL	AUC_0-t_ = 2537 h·μIU/mL	0.005	[32]
	Intranasal insulin	Mouse	2.4 IU	Intranasal administration	AUC_0-t_ = 354 h·μIU/mL	AUC_0-t_ = 3442 h·μIU/mL	9.72	[32]
a-Glucosidase inhibitors	Acarbose	Human	200 mg.	Oral administration	Absorbed only 1–2% of dose	N.D.	N.D.	[33]
	Miglitol	Human	50 mg	Oral administration	AUC_0-t_ = 5998 ng·h/mL	Permeation from BBB is low	N.D.	[34]
Sulfonylureas	Gliclazide	Rat	20 mg/kg	Intraarterial injection	Concentration = 15.99 μg/ml	Concentration = 1.71 µg/g	0.12 g·mL	[35]
	Glibenclamide	Rat	50 mg/kg	Intraperitoneal injection	Concentration = 34 μg/ml	Concentration = 85 ng/ml	0.0025	[36]
Biguanides	Metformin	Rat	150 mg/kg	Oral administration	Concentration = 13.8 µmol/L	Concentration = 13.5 µmol/L	0.99	[37]
Thiazolidinediones	Pioglitazone	Mouse	10.2 mg/kg	Oral administration	N.D.	Concentration = 712.6 pmol/L	N.D.	[38]
	Rosiglitazone	Rodent	Unknown	Unknown	N.D.	0.045% Inject/g tissue	20–30 µL/g *^,†^	[39]
DPP-4 inhibitors	Linagliptin	Rat	2 mg/kg	Intravenous injection	N.D.	BLD	N.D.	[40]
	Omarigliptin	Rat	5 mg/kg	Oral administration	Concentration = 2688.79 ng/mL	Concentration = 621.75 ng/g	0.23 g·mL	[41]
	Trelagliptin	Rat	20 mg/kg	Oral administration	Concentration = 1754.79 ng/mL	N.D.	N.D.	[41]
SGLT2 inhibitors	Ipragliflozin	Mouse	3 mg/kg	Oral administration	AUC_0-t_ = 4520 ng·h/mL	AUC_0-t_ = 2020 ng·h/g	0.5 mL/g	[42]
	Dapagliflozin	Mouse	3 mg/kg	Oral administration	AUC_0-t_ = 2970 ng·h/mL	AUC_0-t_ = 904 ng·h/g	0.3 mL/g	[42]
	Tofogliflozin	Mouse	3 mg/kg	Oral administration	AUC_0-t_ = 1010 ng·h/mL	AUC_0-t_ = 315 ng·h/g	0.3 mL/g	[42]
	Canagliflozin	Mouse	3 mg/kg	Oral administration	AUC_0-t_ = 1620 ng·h/mL	AUC_0-t_ = 532 ng·h/g	0.3 mL/g	[42]
	Empagliflozin	Mouse	3 mg/kg	Oral administration	AUC_0-t_ = 626 ng·h/mL	AUC_0-t_ = 313 ng·h/g	0.5 mL/g	[42]
	Luseogliflozin	Mouse	3 mg/kg	Oral administration	AUC_0-t_ = 478 ng·h/mL	AUC_0-t_ = 157 ng·h/g	0.3 mL/g	[42]
GLP-1 receptor agonists	Exenatide	Mouse	Unknown	Intravenous injection	N.D.	Concentration = 17.8 µL/g	N.D.	[43]
	Liraglutide	Mouse	250 nmol/kg	Intraperitoneal injection	N.D.	Concentration = 200–300 pmol/L *	N.D.	[44]
	Lixisenatide	Mouse	250 nmol/kg	Intraperitoneal injection	N.D.	Concentration = 100–150 pmol/L*	N.D.	[44]
	Semaglutide	Mouse	1 × 10^6^ cpm	Intravenous injection	N.D.	Brain influx rates = N.S.	N.D.	[45]

*: There was no description of the exact value. N.D.: no data. N.S.: not significant. BLD: below limit of detection. ^†^: The value calculated by the following equation: brain–serum ratio = (cpm/brain)/[(cpm/µL serum) · (brain weight)].

## Data Availability

Not applicable.
